# The public health impact of loneliness during the COVID-19 pandemic

**DOI:** 10.1186/s12889-022-14055-2

**Published:** 2022-08-31

**Authors:** James Allen, Oliver Darlington, Karen Hughes, Mark A. Bellis

**Affiliations:** 1 World Health Organization Collaborating Centre On Investment for Health and Well-Being, Public Health Wales, 2 Capital Quarter, Tyndall Street, Cardiff, CF10 4BZ UK; 2 World Health Organization Collaborating Centre On Investment for Health and Well-Being, Public Health Wales, Wrexham, UK; 3 Public Health Collaborating Unit, School of Medical and Health Sciences, Bangor University, Wrexham, UK

**Keywords:** COVID-19, Loneliness, Public health, Mental health, Physical health

## Abstract

**Background:**

Social distancing measures have been effective in mitigating the spread of COVID-19; however, they have imposed a significant burden on population mental health and well-being. This study aimed to identify factors associated with loneliness during the COVID-19 pandemic, and to describe the relationship between loneliness and the risk of worsening health outcomes and behaviours.

**Methods:**

Data for 8,960 adults were drawn from a national cross-sectional survey undertaken in Wales between January and June 2021. Participants self-reported changes to health and behaviour since the start of the pandemic. Logistic regression was used to identify factors associated with loneliness, and the impact of loneliness on self-reported changes in physical health, physical fitness, mental health, weight, alcohol consumption and social relations in comparison with pre-pandemic experiences.

**Results:**

Groups most at risk of loneliness were those aged < 35 years, women (odds ratio [95% confidence interval]: 1.86 [1.70–2.05]), those with chronic health conditions (1.43 [1.29–1.58]) and the unemployed (2.18 [1.76–2.70]). Loneliness was a strong predictor of worsening health outcomes and behaviours, with those reporting often feeling lonely being at increased odds of worsening physical health (3.29 [2.80–3.86]), physical fitness (2.22 [1.90–2.60]), mental health (8.33 [6.95–9.99]), weight (1.39 [1.19–1.62]), alcohol consumption (1.37 [1.12,-1.66]) and social relations (2.45 [2.07–2.89]) during the pandemic.

**Conclusion:**

This study established an association between loneliness and self-reported worsening health during the pandemic, and identified factors increasing the risk of loneliness. The effect that social control measures have on loneliness should influence the design of future public health policy.

**Supplementary Information:**

The online version contains supplementary material available at 10.1186/s12889-022-14055-2.

## Background

The COVID-19 pandemic has had an unprecedented impact on global life. The ease of transmission and virulence of the virus has meant that reducing interactions between people in society has been one of the only ways of controlling its spread, particularly before vaccines became available. Control measures such as social distancing, working from home mandates and business closures have been effective in reducing the direct health impacts of COVID-19 globally, [1, 2] but have also had a dramatic impact on people’s ability to live their lives by limiting the ability to congregate and socialise with colleagues, friends and family.

Although social distancing measures have been effective in mitigating the spread of COVID-19, they are also associated with significant costs. They have had a dramatic impact on global economies by disrupting supply chains and affecting leisure, hospitality and tourism, and economic support packages for badly affected businesses have put a huge strain on countries’ finances [3]. Furthermore, they have also imposed a significant burden on people’s mental health by limiting their ability to socialise; there have been significant increases in the number of people experiencing loneliness and mental health problems in those countries where “lockdowns” have been introduced [4].

The association between loneliness and adverse health outcomes is well established; [5, 6] however, it is often overlooked when considering public health interventions despite having a comparable impact on the risk of negative health outcomes such as smoking and substance misuse [7]. People who regularly experience loneliness have been shown to be at increased risk of depression, anxiety, cardiovascular disease and mortality, and are also more likely to exhibit negative health behaviours which are fundamental in the association between loneliness and poor health outcomes, for example, excess alcohol consumption, smoking and substance abuse [3, 8].

To inform public health interventions in the ongoing COVID-19 pandemic or future epidemic or pandemic events, it is important to understand the broad impact of the COVID-19 pandemic on population health and well-being. As such, the objectives of this study were to assess sociodemographic characteristics of individuals most at risk of experiencing loneliness during the COVID-19 pandemic, and to explore the relationship between loneliness and worsening health and well-being during the pandemic.

## Methods

In April 2020, Public Health Wales (the Welsh national public health agency) initiated a cross-sectional telephone survey to monitor the impact of COVID-19 and related control measures on population health and well-being, in order to inform the national public health response. The survey interviewed around 600 randomly selected adults in Wales each survey week, with surveying initially occurring weekly then, from September 2020, every other week. Sampling and data collection were undertaken by a professional market research company, with landline and mobile telephone contacts obtained from a commercial sample provider. Sampling was stratified by age and gender within Local Authority area to attain a sample broadly representative of the Welsh adult population. On telephone contact, potential participants were provided with a description of the survey including its purpose and voluntary, anonymous and confidential nature, and verbal informed consent was recorded as part of the survey script. Inclusion criteria were aged ≥ 18 years, resident in Wales and cognitively able to participate.

The survey questionnaire (available in Welsh and English) was developed by Public Health Wales and included a set of core questions on COVID-19, related restrictions, health and well-being and participant demographics, with further questions changing to address emergent public health and policy issues. Between 4th January and 24^th^ July 2021 (covering 15 survey rounds), a set of questions was included in the survey measuring changes in health and well-being since the start of the pandemic. This study is based on a secondary analysis of anonymous data from survey participants during this period. A total of 23,672 telephone calls to potential participants were answered during this period, resulting in 13,845 refusals, 856 call-back requests (carried forward to later surveys) and 8,971 completed surveys (38% of answered calls).

A full list of questions used in the study and their post-hoc categorisations are included in the Additional file 1: Supplementary Appendix. Loneliness was measured by a question asking how often participants had felt lonely in the last week. Worsening health, well-being and financial situation during the pandemic was identified through a set of questions asking participants if, compared to a year ago (pre-pandemic), the following were much better, a bit better, the same, a bit worse or much worse: their mental health, their physical fitness, their physical health, their social relations, their financial situation; and over the same time period, whether the following had increased, stayed the same or reduced: their alcohol consumption, their weight. Having a pre-existing health condition (diabetes, heart disease, lung disease or cancer) and having personally had COVID-19 were self-reported.

Postcode of residence was categorised into deprivation quintile by the market research company using the Welsh Index of Multiple Deprivation (WIMD) [9]. Age was categorised into four groups (18–34 years, 35–54 years, 55–74 years, ≥ 75 years). Ethnicity was self-defined using UK census categories, but due to low levels in non-white categories was re-categorised to Minority ethnic group, yes or no. Employment status was re-categorised into employed, unemployed and other. For the purpose of analysis, gender was restricted to male and female due to very low numbers responding transgender or other/prefer not to say. Thus, the final sample size for analysis was 8,960.

### Statistical analysis

Statistical differences in loneliness between groups were initially assessed using Pearson’s χ^2^ test. A logistic regression model of complete cases was used to identify independent associations (adjusted odds ratios [AORs] and 95% confidence intervals [CIs]) between respondent characteristics and experiencing loneliness. Additional logistic regression models of complete cases were developed to measure the relationship between loneliness and worsening health outcomes and behaviours. These models were adjusted for individual demographic and economic characteristics of the respondent. All statistical analysis assumed a significance threshold of α = 0.05. Stata 14 was used to conduct statistical analysis.

## Results

### Characteristics of survey participants

Participant characteristics are presented in Table 1. Half of respondents (49.6%) were aged between 55 and 74 and a third were aged 35–54 (27.7%), with smaller proportions in the youngest (18–34, 9.6%) and oldest (≥ 75, 13.1%) age groups. Only 2.4% of the sample was categorised as being from a minority ethnic group. The sample was evenly distributed across deprivation quintiles (ranging between 17.2% in the most deprived to 21.9% in the least deprived quintile). Over a quarter of respondents (28.3%) reported having a chronic health condition and 16.1% believed they had been infected with COVID-19. Just over half were employed (50.5%) at the time of the survey and two thirds (66.4%) reported no change in their financial situation compared to their situation prior to the COVID-19 pandemic.

**Table 1:** Participant demographics and bivariate relationships with loneliness in the last week

	**Sample** n (%)	**In the last week, how often have you felt lonely (%)**					
		**Never**	**Occasionally**	**Often**	**Always**	${{\varvec{\chi}}}^{2}$	***p***
**All**	8,960 (100.0)	62.9	24.7	9.0	3.5		
**Age group (years)**							
18–34	861 (9.6)	49.6	31.7	14.3	4.4	95.7	< 0.001
35–54	2,482 (27.7)	61.8	24.7	9.9	3.6		
55–74	4,447 (49.6)	65.2	23.3	8.1	3.4		
≥ 75	1,170 (13.1)	66.0	24.7	6.5	2.8		
**Sex**							
Female	5,443 (60.7)	57.3	28.0	10.7	4.0	188.7	< 0.001
Male	3,517 (39.3)	71.5	19.5	6.3	2.6		
**Minority ethnic group**							
No	8,748 (97.6)	63.1	24.6	9.0	3.3	18.8	< 0.001
Yes	212 (2.4)	52.8	29.7	9.4	8.0		
**Chronic health condition**^a^							
No	6,428 (71.7)	64.9	24.3	8.1	2.7	74.2	< 0.001
Yes	2,532 (28.3)	57.7	25.6	11.3	5.4		
**Had COVID-19**							
No	7,079 (79.0)	63.4	24.8	8.5	3.3	23.4	0.005
Yes, and now recovered	1,208 (13.5)	62.8	22.8	10.5	3.8		
Yes, and currently has symptoms	233 (2.6)	52.8	30.0	12.0	5.2		
Don't know	440 (4.9)	58.9	25.7	10.9	4.5		
**Employment status**							
Employed	4,529 (50.5)	65.5	24.7	7.9	2.0	192.6	< 0.001
Unemployed	425 (4.7)	38.8	33.9	17.4	9.9		
Other^b^	4,006 (44.7)	62.4	23.7	9.4	4.5		
**Financial situation**							
The same	5,947 (66.4)	65.6	23.7	7.5	3.2	413.5	< 0.001
Much better	404 (4.5)	69.8	20.3	7.4	2.5		
A bit better	1,036 (11.7)	68.7	24.9	5.6	0.8		
A bit worse	1,005 (11.2)	50.1	32.0	13.5	4.3		
Much worse	568 (6.3)	40.5	25.2	23.8	10.6		
**Deprivation quintile**							
1—Most deprived	1,538 (17.2)	56.2	26.3	11.7	5.9	110.4	< 0.001
2	1,827 (20.4)	59.7	26.3	9.0	5.0		
3	1,747 (19.5)	63.4	24.6	9.3	2.7		
4	1,888 (21.1)	67.3	23.3	7.3	2.2		
5—Least deprived	1,960 (21.9)	66.3	23.5	8.2	2.0		

^a^including diabetes, heart disease Diabetes, lung disease (e.g. asthma, COPD) or cancer

^b^includes students, long-term sick or disabled, those not working for domestic reasons and other

### Relationships between sociodemographic characteristics and loneliness

Reported loneliness in the week prior to survey varied considerably by demographics (Table 1). The proportion never feeling lonely increased with age, rising from 49.6% of 18–34 year olds to 66.0% of those aged ≥ 75. A greater proportion of males (71.5%) reported never feeling lonely than females (57.3%) while a smaller proportion of respondents who were unemployed reported never feeling lonely (38.8%) compared to other groups. When looking at different levels of loneliness, more frequent loneliness tended to be higher in females, younger age groups, minority ethnic groups, the unemployed and those living in more deprived areas, with significantly different distributions across the different levels of loneliness.

**Fig. 1:**
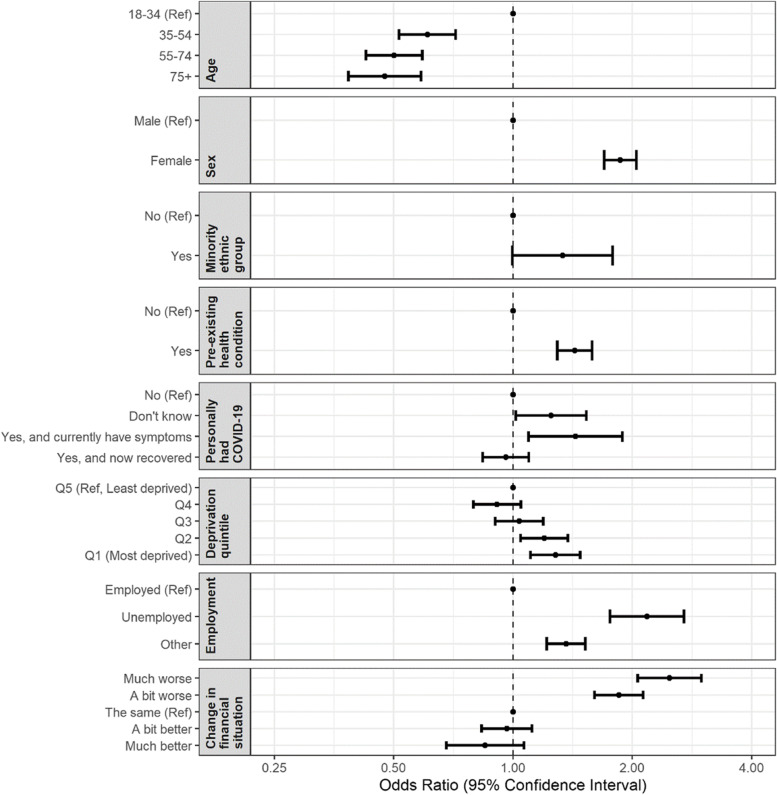
Adjusted odds of reporting a feeling of loneliness in the last week* by socio-demographic characteristics *Loneliness defined as those reporting feeling ‘Occasionally’, ‘Often’ or ‘Always’ lonely, in the last week

In a multivariable model (Fig. 1), people aged 35 and over were found to have significantly decreased odds of reporting loneliness (occasionally, often or always) compared with those aged 18–34 years (35–54 years, AOR 0.61 [95% CI: 0.52–0.72], 55–74 years, 0.50 [0.43–0.59], ≥ 75 years, 0.47 [0.38–0.59]). Females (1.86 [1.70–2.05]), those with chronic health conditions (1.43 [1.29–1.58]) those who reported currently having COVID-19 symptoms (1.44 [1.09–1.89]) and those who were unemployed (2.18 [1.76–2.70]) were at significantly increased odds of reporting feeling lonely. Financial situation was a strong predictor of loneliness, with those who reported their financial situation to have worsened over the pandemic being at significantly increased odds of loneliness (‘A bit worse’, 1.85 [1.61–2.13], ‘Much worse’, 2.48 [2.06–2.99]). Similarly, respondents in the more deprived quintiles were at significantly increased odds of loneliness compared with respondents in the least deprived quintile (e.g. most deprived quintile, 1.28 [1.11–1.48]).

### Relationships between loneliness and negative health behaviours and outcomes

Loneliness was independently associated with all health outcomes measured (Table 2). Compared with those who reported never feeling lonely; those reporting any level of loneliness were at around 30% greater odds of reporting both increased alcohol consumption and increased weight during the pandemic. For other outcomes, odds increased with the severity of loneliness. Thus, odds of reporting worsening physical fitness increased from 1.58 [95% CI: 1.43–1.76] for those who felt lonely occasionally to 2.82 [2.20–3.62] for those who always felt lonely (v those never feeling lonely). Similarly, odds of worsening physical health increased from 1.81 (1.63–2.01; occasionally lonely) to 3.57 (2.77–4.59; always lonely). For worsening social relationships, odds increased from 1.73 [1.56–1.93] for occasional loneliness to around 2.4 for those feeling always or often lonely (Table 2).

**Table 2:** Adjusted odds ratios for worsening physical, mental and behavioural outcomes during the pandemic

	**Respondents who reported a worsening in health outcomes and behaviours, compared to this time a year ago**											
	**Worse mental health**		**Worse physical fitness**		**Worse physical health**		**Worse social relations**		**Increased alcohol consumption**		**Increased weight**	
	AOR (95% CI)		AOR (95% CI)		AOR (95% CI)		AOR (95% CI)		AOR (95% CI)		AOR (95% CI)	
**Loneliness (last week)**												
Never lonely	Ref		Ref		Ref		Ref		Ref		Ref	
Occasionally lonely	3.20*	(2.87—3.57)	1.58*	(1.43—1.76)	1.81*	(1.63—2.01)	1.73*	(1.56—1.93)	1.32*	(1.15—1.50)	1.34*	(1.21—1.48)
Often lonely	8.33*	(6.95—9.99)	2.22*	(1.90—2.60)	3.29*	(2.80—3.86)	2.45*	(2.07—2.89)	1.37*	(1.12—1.66)	1.39*	(1.19—1.62)
Always lonely	8.03*	(6.09—10.60)	2.82*	(2.20—3.62)	3.57*	(2.77—4.59)	2.41*	(1.86—3.11)	1.39*	(1.02—1.91)	1.29*	(1.02—1.64)
**Age group (years)**												
18–34	Ref		Ref		Ref		Ref		Ref		Ref	
35–54	1.35*	(1.13—1.61)	1.33*	(1.13—1.57)	1.51*	(1.27—1.79)	1.39*	(1.18—1.64)	1.31*	(1.08—1.60)	1.19*	(1.02—1.40)
55–74	0.87	(0.73—1.04)	1.04	(0.88—1.22)	1.12	(0.94—1.32)	1.27*	(1.08—1.49)	0.93	(0.76—1.14)	1.06	(0.90—1.24)
≥ 75	0.45*	(0.35—0.57)	0.99	(0.80—1.22)	1.12	(0.90—1.39)	0.79*	(0.64—0.97)	0.55*	(0.41—0.75)	0.79*	(0.64—0.98)
**Sex**												
Male	Ref		Ref		Ref		Ref		Ref		Ref	
Female	1.53*	(1.38—1.69)	1.34*	(1.22—1.46)	1.24*	(1.13—1.36)	1.09	(1.00—1.19)	1.05	(0.94—1.19)	1.75*	(1.59—1.91)
**Minority ethnic group**												
No	Ref		Ref		Ref		Ref		Ref		Ref	
Yes	0.67*	(0.49—0.92)	0.89	(0.66—1.18)	1.06	(0.79—1.43)	0.66*	(0.50—0.88)	0.51*	(0.33—0.79)	0.88	(0.66—1.18)
**Chronic health condition**^a^												
No	Ref		Ref		Ref		Ref		Ref		Ref	
Yes	1.21*	(1.08—1.35)	1.44*	(1.31—1.60)	3.29*	(2.80—3.86)	0.89*	(0.80—0.98)	0.75*	(0.65—0.86)	1.12*	(1.01—1.23)
**Had COVID-19**												
No	Ref		Ref		Ref		Ref		Ref		Ref	
Yes, and now recovered	1.30*	(1.13—1.50)	1.45*	(1.28—1.65)	1.48*	(1.30—1.69)	1.02	(0.90—1.16)	1.05	(0.89—1.23)	1.10	(0.97—1.25)
Yes, and currently have symptoms	1.79*	(1.33—2.40)	2.49*	(1.88—3.29)	3.79*	(2.83—5.08)	1.07	(0.81—1.41)	0.76	(0.52—1.11)	1.11	(0.85—1.46)
Don't know	1.24	(1.00—1.54)	1.27*	(1.04—1.55)	1.37*	(1.12—1.69)	1.14	(0.93—1.40)	0.90	(0.69—1.17)	1.07	(0.88—1.31)
**Employment status**												
Employed	Ref		Ref		Ref		Ref		Ref		Ref	
Unemployed	0.89	(0.71—1.12)	0.98	(0.79—1.21)	0.88	(0.71—1.10)	0.70*	(0.57—0.87)	0.69*	(0.52—0.91)	0.96	(0.78—1.19)
Other^b^	0.95	(0.84—1.07)	1.15*	(1.03—1.28)	1.55*	(1.40—1.72)	0.83*	(0.75—0.93)	0.67*	(0.58—0.78)	0.87*	(0.78—0.98)
**Financial situation**												
The same	Ref		Ref		Ref		Ref		Ref		Ref	
Much better	0.88	(0.70—1.12)	0.83	(0.66—1.03)	0.95	(0.76—1.20)	0.98	(0.80—1.21)	1.26	(0.97—1.64)	0.82	(0.66—1.02)
A bit better	1.14	(0.98—1.33)	1.09	(0.95—1.25)	0.98	(0.84—1.13)	1.01	(0.88—1.16)	1.30*	(1.10—1.54)	0.97	(0.84—1.11)
A bit worse	2.12*	(1.82—2.47)	1.47*	(1.28—1.69)	1.54*	(1.34—1.78)	1.32*	(1.14—1.53)	0.99	(0.82—1.19)	1.05	(0.92—1.21)
Much worse	3.54*	(2.87—4.38)	1.92*	(1.59—2.32)	2.19*	(1.81—2.65)	1.68*	(1.38—2.04)	1.44*	(1.16—1.79)	1.24*	(1.04—1.49)
**Deprivation quintile**												
5—Least deprived	Ref		Ref		Ref		Ref		Ref		Ref	
4	0.88	(0.76—1.02)	0.91	(0.80—1.05)	0.93	(0.81—1.07)	0.92	(0.81—1.05)	0.88	(0.75—1.04)	0.97	(0.85—1.11)
3	0.86	(0.74—1.00)	0.89	(0.78—1.02)	0.93	(0.81—1.08)	0.82*	(0.71—0.93)	0.70*	(0.59—0.83)	0.93	(0.81—1.06)
2	0.82*	(0.71—0.96)	1.05	(0.91—1.20)	1.08	(0.94—1.24)	0.82*	(0.72—0.94)	0.70*	(0.59—0.83)	1.03	(0.90—1.17)
1—Most deprived	0.82*	(0.70—0.96)	1.10	(0.95—1.26)	1.18*	(1.02—1.37)	0.79*	(0.69—0.91)	0.64*	(0.53—0.77)	1.09	(0.95—1.26)

^a^including diabetes, heart disease, lung disease (e.g. asthma, COPD) or cancer

^b^includes students, long-term sick or disabled, those not working for domestic reasons and other

^*^difference is statistically significant

The association between loneliness and mental health was particularly strong. Odds of reporting worsening mental health were 3.20 [2.87–3.57] even in those reporting occasionally feeling lonely and increased to 8.33 [6.95–9.99] and 8.03 [6.09–10.60] in those feeling often and always lonely respectively.

## Discussion

Using a large national sample, our study has identified significant associations between loneliness and worsening physical and mental health during the COVID-19 pandemic. Over a third (37.2%) of respondents reported feeling lonely in the last week, with groups at increased risk of experiencing loneliness cutting across multiple demographic characteristics, including young people, women, those with pre-existing health conditions, people living in more deprived areas, the unemployed, and those experiencing a negative change in their financial situation. Loneliness was the strongest predictor of reporting worsening mental health, with people who reported having often or always felt lonely in the last week having over an eight-fold increase in odds of self-reporting their mental health to have worsened during the pandemic. This effect was also observed with respect to physical health outcomes, with odds of reporting worsening physical health and physical fitness being over three-fold and two-fold respectively for those always or often lonely.

The relationships identified here between loneliness and worsening physical and mental health are consistent with those found in previous studies conducted prior to the pandemic in effect size [5–7]. Our analysis has additionally shown that these relationships are maintained in the context of the COVID-19 pandemic. This is of particular concern during the pandemic due to the reduced ability for people to socialise with friends and family, increasing the number of people experiencing loneliness as a result [10, 11] and potentially translating to a substantial indirect effect on public health.

Our study also highlights the potential for lasting negative health consequences associated with the COVID-19 pandemic, with 40.1% of people reporting increased weight, and 16.9% of people reporting increased alcohol consumption in comparison with pre-pandemic. These poorer health behaviours were more prevalent in people who reported feeling often or always lonely, with a 37% and 29% increase in the odds of reporting increased alcohol consumption and increased weight in comparison with pre-pandemic, respectively. These observations may partially mediate the wider relationship between loneliness and physical health outcomes.

No other factors were found to have as consistent or strong a relation with the odds of all self-reported poor physical, mental and health behaviour outcomes as loneliness, although people who described their financial situation as much worse in comparison with prior to the COVID-19 pandemic were consistently at increased risk of poorer outcomes. For example, people who reported being in a ‘Much worse’ financial situation compared to a year ago were over three times more likely to report worsening mental health and over two times likely to report worse physical health.

Our analysis suggests that the social impact of the COVID-19 pandemic may also have the effect of compounding pre-existing health inequalities present in society. Previous studies have shown the disproportionate direct effect of COVID-19 on people living in more deprived areas and on those from a minority ethnic background [12, 13]. This study has shown that in addition to these direct effects, people from these population groups are also significantly more likely to report feeling often or always lonely, and may be at increased risk of worsening physical, mental and behaviour health outcomes as a result.

The observed relationships in this study may also have a downstream effect on health service use; not only the demand for mental health support services indicated by the increased odds of reporting worse mental health and social isolation, but also the demand for services treating clinical outcomes relating to increased weight, increased alcohol consumption and worse physical health and fitness. In addition, these services are often under increased pre-existing pressure in more deprived areas, potentially further exacerbating health inequities for the most deprived [14, 15].

A primary limitation of this analysis is that all outcomes were based on self-reported data; as such a respondent’s current mental health may affect their perception of their current physical health, and vice versa. The use of a self-rating question to measure loneliness could lead to under-reporting in certain population groups, for example, there is evidence to suggest under-reporting of loneliness in men when using a direct measure (as used in this study) compared to the use of an indirect measure such as the De Jong Gierveld Scale [16]. All data was cross-sectional rather than longitudinal meaning that causative relationships could not be measured between loneliness and the examined outcomes. Loneliness was measured in the past week and changes in health over the past year. It was not possible to account for changes in individuals’ perceptions of their mental or physical health over time. Results may also be confounded by unobserved data. No analysis was undertaken on separate ethnic groups due to the small sample size and only 2.4% of the sample were from a minority ethnic group, which may limit the generalisability of the study findings with respect to ethnic background. There are also limitations based on the administration of the survey; as this analysis is based on a sample of data from respondents who agreed to participate in a telephone survey, results may be subject to selection bias and may not be generalizable to the population as a whole.

## Conclusion

We have identified population groups who were at increased risk of experiencing loneliness during the COVID-19 pandemic, and the association between loneliness and self-reported worsening physical and mental health, as well as negative health behaviours. The adoption of such behaviours is likely to play an important role connecting loneliness to poorer physical and mental health [8]. Our analysis indicates that women, young people, people with a black or minority ethnic background, those with pre-existing chronic health conditions and people living in more deprived areas were more likely to report feeling lonely throughout the pandemic. Previous studies have shown that these groups are likely to experience the worst outcomes directly due to COVID-19 infection, [12, 13, 17] and our analysis suggests that they may also be at the most risk of indirect effects due to social distancing measures. Further, with the potential long-term effects of health behaviours such as increased alcohol consumption and reduced physical activity, the impact of the COVID-19 pandemic on health measures may not yet be fully realised. Should further social distancing measures be required in the future, this analysis indicates the potential public health benefits of considering these societal groups for a targeted policy response and health service provision. Although vaccines for COVID-19 are now available, in some areas and countries uptake remains low. The risk of new pandemics emerging will never recede, and as new variants of COVID-19 with the potential to affect vaccine efficacy emerge, there may be new requirements for non-pharmaceutical interventions such as lockdowns to be imposed by governments in the future. The potential impact of these interventions on loneliness should influence the design and implementation of future public health policy.

## Supplementary Information

**Additional file 1: Supplementary table 1. **Variables selected from the cross-sectional and post-hoc categorisation for analysis

## Data Availability

The datasets analysed during the current study is available from the corresponding author on reasonable request.
