# Supplementary material for: The public health impact of loneliness during the COVID-19 pandemic
Source: BMC Public Health. 2022 Aug 31;22:1654. doi: 10.1186/s12889-022-14055-2 (PMC9433133; doi:10.1186/s12889-022-14055-2)
Supplement: Supplementary file 1 — Additional file 1: Supplementary table 1. Variables selected from the cross-sectional and post-hoc categorisation for analysis [file 12889_2022_14055_MOESM1_ESM.docx]

**Additional file 1**

**Supplementary table 1** Variables selected from the cross-sectional and post-hoc categorisation for analysis

| **Variable** | **Survey question** | **Value** | **Value label** | **Analysis value** |
| --- | --- | --- | --- | --- |
| Age | Can I ask what your year of birth is? | 1 | 18-34 | 1 |
|  |  | 2 | 35-54 | 2 |
|  |  | 3 | 55-74 | 3 |
|  |  | 4 | ≥75 | 4 |
|  | | | | |
| Gender | What is your gender? | 1 | Male | 1 |
|  |  | 2 | Female | 2 |
|  |  | 3 | Transgender | - |
|  |  | 4 | Other/Prefer not to say | - |
|  |  |  |  |  |
| Ethnicity | Minority Ethnic Group – known to be from BAME, missing or prefer not to say | 0 | No | 0 |
|  |  | 1 | Minority ethnic group | 1 |
|  |  |  |  |  |
| Pre-existing health condition | Please could you tell me if you have any of these conditions? Diabetes, heart disease, lung disease (e.g. asthma, COPD) or cancer? INT: Code one | 0 | No | 0 |
|  |  | 1 | Yes | 1 |
|  |  |  |  |  |
| Personally had COVID-19 | Do you think you have or have had coronavirus? | 1 | Yes, and now recovered | 1 |
|  |  | 2 | Yes, and currently have symptoms | 1 |
|  |  | 3 | No | 0 |
|  |  | 4 | Don’t know | 0 |
|  |  |  |  |  |
| Welsh Index of Multiple Deprivation quintile | Postcode of respondent | 1 | Most deprived | 1 |
|  |  | 2 | 2 | 2 |
|  |  | 3 | 3 - Middle | 3 |
|  |  | 4 | 4 | 4 |
|  |  | 5 | Least deprived | 5 |
|  |  |  |  |  |
| Employment | What is your current employment status? | 1 | Employed full time (35+ hours per week) | 1 |
|  |  | 2 | Employed part time (less than 35 hours per week) | 1 |
|  |  | 3 | Self-employed | 1 |
|  |  | 4 | Student | 3 |
|  |  | 5 | Unemployed | 2 |
|  |  | 6 | Long-term sick or disabled | 3 |
|  |  | 7 | Retired | 3 |
|  |  | 8 | Carer or not working for domestic reasons | 3 |
|  |  | 9 | Other | 3 |
|  |  |  |  |  |
| Change in financial situation | Compared to this time a year ago, would you say the following are much better, a bit better, the same, a bit worse or much worse…? Your financial situation | 1 | Much better | 1 |
|  |  | 2 | A bit better | 2 |
|  |  | 3 | The same | 3 |
|  |  | 4 | A bit worse | 4 |
|  |  | 5 | Much worse | 5 |
|  |  |  |  |  |
| Worse mental health | Compared to this time a year ago, would you say the following are much better, a bit better, the same, a bit worse or much worse…? Your mental health | 1 | Much better | 0 |
|  |  | 2 | A bit better | 0 |
|  |  | 3 | The same | 0 |
|  |  | 4 | A bit worse | 1 |
|  |  | 5 | Much worse | 1 |
|  |  |  |  |  |
| Worse physical fitness | Compared to this time a year ago, would you say the following are much better, a bit better, the same, a bit worse or much worse…? Your physical fitness | 1 | Much better | 0 |
|  |  | 2 | A bit better | 0 |
|  |  | 3 | The same | 0 |
|  |  | 4 | A bit worse | 1 |
|  |  | 5 | Much worse | 1 |
|  |  |  |  |  |
| Worse physical health | Compared to this time a year ago, would you say the following are much better, a bit better, the same, a bit worse or much worse…? Your physical health | 1 | Much better | 0 |
|  |  | 2 | A bit better | 0 |
|  |  | 3 | The same | 0 |
|  |  | 4 | A bit worse | 1 |
|  |  | 5 | Much worse | 1 |
|  |  |  |  |  |
| Worse social relations | Compared to this time a year ago, would you say the following are much better, a bit better, the same, a bit worse or much worse…? Your social relations | 1 | Much better | 0 |
|  |  | 2 | A bit better | 0 |
|  |  | 3 | The same | 0 |
|  |  | 4 | A bit worse | 1 |
|  |  | 5 | Much worse | 1 |
|  |  |  |  |  |
| Increased alcohol consumption | Compared to this time a year ago, would you say the following have reduced, stayed the same or increased…? Your alcohol consumption | 1 | Reduced | 0 |
|  |  | 2 | Stayed the same | 0 |
|  |  | 3 | Increased | 1 |
|  |  |  |  |  |
| Increased weight | Compared to this time a year ago, would you say the following have reduced, stayed the same or increased…? Your weight | 1 | Reduced | 0 |
|  |  | 2 | Stayed the same | 0 |
|  |  | 3 | Increased | 1 |
|  |  |  |  |  |
| Loneliness | In the last week, how often have you felt lonely? You can say 'never', 'occasionally', 'often' or 'always'... | 1 | Never | 1 |
|  |  | 2 | Occasionally | 2 |
|  |  | 3 | Often | 3 |
|  |  | 4 | Always | 4 |
